# The Effect of Calcium, Water, and Other Substances Given Intravenously on Blood Composition and Urinary Secretion

**Published:** 1917-04

**Authors:** D. M. Davis


### The Effect of Calcium, Water, and Other Substances Given Intravenously on Blood Composition and Urinary Secretion.

BY D. M. DAVIS.

Experiments are described in which it was desired to inject quantities of fluid intravenously in animals without consequent diuresis. This end was attained by injecting distilled water, mixtures of $CaCl_2$ with NaCl solutions, and dilute solutions of dextrose at such rates that the animal received less than .85 gms. dextrose per kilogram hour.

Overdoses of Ca made the blood become more concentrated, with increased hemoglobin and diminished water content.

In all the other injections, there was definite hydremia, as shown by the dried weight of the blood.

It appears that diuresis may be absent, therefore, while hydremia is present, and also while the blood sugar is increased by over 100 per cent. Since the $\Delta$ has usually shown a fall, the author hazards the suggestion that the tendency to diuresis, which is the result of an increase of any excretable substance in the blood may be counteracted by a simultaneous decrease of some other excretable substance in the blood.

Other aspects of the theory of diuresis are discussed.

The inhibitory action of Ca on urinary secretion, shown by J. B. MacCallum, is confirmed.

---

There is no Federal institution in the continental United States for the reception and care of lepers.

---

Patient's Wife—"I hope you gentlemen are not going to disagree!"

First Doctor—"Oh, not about anything important. Dr. Fell insists on trepanning; Dr. Fluke holds out for removing the appendix, and I am in favor of amputation at the knee. But we're all agreed that on operation is necessary."—*Life.*

---

Temperature Wasn't Right.—An ignorant fellow had been persuaded to buy a thermometer by a glib-tongued salesman, and a few days later he came back with it, complaining that it didn't give satisfaction.

"What's the matter with it?" asked the clerk.

"Ah dunno, but it ain't made no diff-rence round mah place. Some days de house am too cold an' odder days it's too hot."

